# The Correlation between Polybrominated Diphenyl Ethers (PBDEs) and Thyroid Hormones in the General Population: A Meta-Analysis

**DOI:** 10.1371/journal.pone.0126989

**Published:** 2015-05-18

**Authors:** Xuemin Zhao, Hailong Wang, Jing Li, Zhongyan Shan, Weiping Teng, Xiaochun Teng

**Affiliations:** 1 Department of Endocrinology and Metabolism, Institute of Endocrinology, The First Affiliated Hospital, China Medical University, Liaoning Provincial Key Laboratory of Endocrine Diseases, Shenyang, P. R. China; 2 Department of Clinical Epidemiology, Institute of Cardiovascular Disease and Center of Evidence Based Medicine, The First Affiliated Hospital, China Medical University, Shenyang, P. R. China; Hokkaido University, JAPAN

## Abstract

**Objective:**

Certain epidemiological studies have suggested exposure to polybrominated diphenyl ethers (PBDEs) affect the production and secretion of thyroid hormones (TH); however, conflicting results have been reported in different studies. There is not a convincing conclusion about this debate to date.

**Materials and Methods:**

To perform a meta-analysis determining if there are correlations between PBDEs exposure and the serum levels of TH. Medical and scientific literature databases were searched for articles that met the eligibility criteria. The included articles were assessed for methodological quality. The correlation coefficient values or regression coefficient values between PBDEs and thyroid stimulating hormone (TSH) or total thyroxine (TT4) from each article were used for analysis.

**Data Synthesis:**

Sixteen articles were included in this meta-analysis. Pearson correlation coefficients (r) were directly collected or calculated from data given in the articles. Then, Fisher’s z transformation was performed to convert each correlation coefficient to an approximately normal distribution. For z values between PBDEs exposure and TSH levels, the pooled z value for 18 studies was 0.08 (95% CI: -0.06, 0.22), and indicated significant heterogeneity (I^2^ values = 90.7%). Subgroup analysis was performed based on the median values of serum PBDEs in each study, there was not significant heterogeneity in each of the four subgroups (I^2^ values <30%). In meta-analysis of z values between PBDEs exposure and the levels of TT4, the pooled z value for 11 studies was -0.02 (95% CI: -0.11, 0.08), and also indicated significant heterogeneity (I^2^ values = 57.6%). Similar subgroup analysis was done for the PBDEs exposures and the levels of TT4. No significant heterogeneity was shown in either of the two subgroups (I^2^ values = 0).

**Conclusion:**

The findings in our meta-analysis indicate the effects of PBDEs on thyroid function may mainly depend on PBDEs exposure and their levels found in serum. The relationship between PBDEs exposure and changes in thyroid function seem to fit an approximate u-shaped curve. These predictions await further verification, namely a prospective longitudinal study.

## Introduction

Polybrominated diphenyl ethers (PBDEs), a class of halogenated chemicals, were used extensively as flame retardants in electronic appliances, textiles, and furnishings, among other goods. Presently, PBDEs are considered ubiquitous environmental contaminants due to their persistence and high production [1–2]. The presence of PBDEs is commonly detected in human tissues and environmental samples worldwide [3–6]. Classic characteristics of PBDEs are bioaccumulation and lipophilicity, and there are 209 congeners, such as PBDE-47, PBDE-99, and PBDE-209 [7–8]. The chemical structures of PBDEs are similar to that of thyroid hormones. Through laboratory animal studies, PBDEs have been shown to be endocrine disrupters, affecting thyroid regulation multiple ways [9–10]. There has not been a consistent conclusion regarding a correlation between PBDEs exposures and TH from epidemiological studies, however. The primary aim of this meta-analysis is to explore the sources of heterogeneity in different studies.

## Material and Methods

### Protocol and registration

The protocol for this meta-analysis was registered in PROSPERO (http://www.crd.york.ac.uk/prospero/) (number: CRD42014013289).

### Search strategy

**Fig 1:**
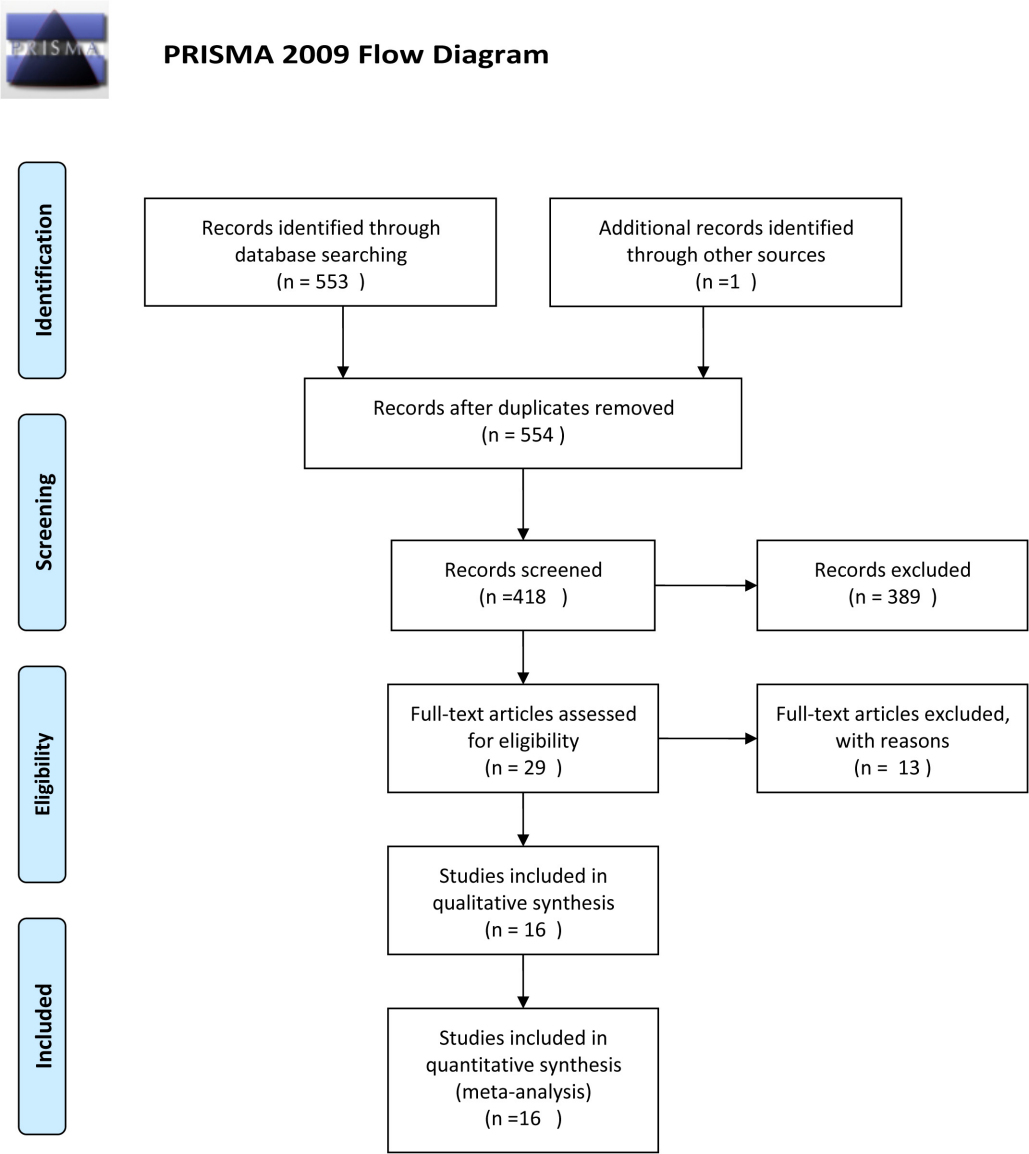
Flowchart of the study’s selection process.

The following databases were independently searched by two researchers in September 2014: PubMed, the Cochrane library, Embase, and China National Knowledge Infrastructure (CNKI). Researchers searched databases for articles published before September 2014 that contained the following terms: polybrominated diphenyl ethers, PBDEs, thyroid hormones, and PBB. The search strategy in PubMed was published in PROSPERO. The screening was initially centered on independent reaches of titles and abstracts (Fig 1). Next, potential articles were sequentially evaluated by full-text reading according to the given criteria.

### Selection criteria

Articles fulfilling the following criteria were included: (1) investigated the associations between PBDEs exposures and thyroid function in the general population (2) the Pearson correlation coefficient (r), Spearman correlation coefficient (r_s_), or regression coefficient (b) were given in the article. Articles fulfilling the following criteria were excluded: (1) studies using animal or in vitro models, (2) studies where the evaluated correlation coefficient (r) could not be acquired, (3) studies where a thyroid disease could impact thyroid hormone secretions, (4) the levels of PBDEs were not acquired from blood.

### Data collection

The data from the included articles were independently extracted by two researchers. The general information for each article; authors, year of publication, number of participants, nation the study was performed in, the median of PBDEs in each study, r_s_ values, r values, b values, and 95% confidence intervals (CIs) of b values are given in Table 1.

**Table 1:** Characteristics of the included studies.

Author	Year	N	Age	Gender	TSH/TT4	MPPB	Nations	PBDEs-TSH				PBDEs-TT4			
								b value	95% CI	r_s_	r	b value	95% CI	r_s_	r
Chevrier	2010	270	A	F	PB-G	25.2	America	-0.08^a^	(-0.14, -0.01)^a^			-0.18^a^	(-0.65, 0.30)^a^		
								-0.07^b^	(-0.13, 0.00)^b^			-0.15^c^	(-0.60, 0.30)^c^		
Kim-1	2009	108	N	F/M	CB	<30^d^	Korea			-0.118^a^					
Lin	2011	54	N	F/M	CB	<30^d^	China			-0.119^a^					-0.091^a^
										-0.2^b^					-0.062^c^
Turyk	2008	304/307	A	M	PB	38.4	America				-0.1^a^				0.12^a^
															0.09^c^
Han-1	2010	108	C	F/M	PB	664	China			0.392^a^					
Han-2	2010	123	C	F/M	PB	375	China			0.783^a^					
Stapleton	2011	137	A	F	PB-G	36.56	America	0.06^a^	(-0.04, 0.17)^a^	0.11^a^		0.5^a^	(0.06, 0.94)^a^	0.21^a^	
										0.14^b^				0.20^c^	
Huang	2014	124	A	F/M	PB	7.16	China			-0.096^a^					
										-0.252^b^					
Kim-2	2012	21	N	F/M	CB	<30^d^	Korea			0.247^a^					
Chevrier	2011	289	N	F/M	PB	25.4	America	0^a^	(-0.06, 0.06)^a^						
								-0.01^b^	(-0.07, 0.04)^b^						
Abdelouahab-1	2013	260	A	F	PB-G	30.92	Canada	0.03^a^	(-0.06, 0.12)^a^			-0.36^a^	(-0.56, -0.13)^a^		
								0.05^b^	(-0.01, 0.07)^b^			-0.29^c^	(-0.51, -0.08)^c^		
Abdelouahab-2	2013	260	A	F	PB-D	30.92	Canada	0.01^a^	(-0.15, 0.18)^a^			-0.06^a^	(-0.33, 0.19)^a^		
								-0.08^b^	(-0.25, 0.09)^b^			0.13^c^	(-0.15, 0.40)^c^		
Abdelouahab-3	2013	260	N	F/M	CB	30.92	Canada	-0.04^a^	(-0.12, 0.04)^a^			-0.35^a^	(-0.61, -0.08)^a^		
								0.03^b^	(-0.04, 0.1)^b^			-0.23^c^	(-0.49, 0.03)^c^		
Zota	2011	25/24	A	F	PB-G	82.9	America	0.4^a^	(-0.07, 0.87)^a^			0.5^a^	(-1.04, 2.04)^a^		
								0.24^b^	(-0.12, 0.6)^b^			0.02^c^	(-1.37, 1.41)^c^		
Zhang	2009	50	A	F	PB-G	<30^d^	China							-0.129^a^	
Xu	2014	45	C	F/M	PB	<30	China			-0.23^a^				0.03^a^	
Kim-3	2012	12	A	F	PB-D	14	Korea			0.517^a^					
Bloom	2008	36	A	F/M	PB	15	America	-0.013^a^	(-0.183, 0.158)^a^						
								-0.006^b^	(-0.273, 0.26)^b^						
Kim-4	2013	105	A	F	PB-G	2.13	Korea	-0.055^a^	(-0.318, 0.209)^a^			0.007^a^	(-0.032, 0.04)^a^		
												0.006^c^	(-0.021, 0.033)^c^		

Abbreviations: M, male; F, female; I, infant; C, child; A, adult; N, neonate; TSH, thyroid stimulating hormone; TT4, total thyroxine; PBDEs, sum of PBDE congeners; PB, samples collected from peripheral blood; CB, samples collected from cord blood; G, samples collected during gestation; D, samples collected after delivery; r, pearson correlation coefficient; r_s_, spearman correlation coefficient; b value, regression coefficient; 95%CI, 95% confidence interval(CI) of b value; MPPB, Median serum PBDEs levels in peripheral blood (ng/g lipid).

^a^ data for the correlations between PBDEs and thyroid function.

^b^data for the correlations between PBDE-99 and thyroid function.

^c^data for the correlations between PBDE-47 and thyroid function.

^d^MPPB estimated by the serum median PBDEs levels in cord blood.

### Assessment of bias risk

Three researchers independently assessed the included articles' methodology quality, using cross-sectional assessment provided by Agency for Healthcare Research and Quality [11]. Here, eleven items were used to assess quality. If the article conformed to the item, we scored it 2. If we cannot assure whether the article conformed to the item, we scored it 1. Of the articles assessed, 4 articles scored in the 18 to 22 range, 7 articles scored in the 13 to 17 range, 5 articles scored in the 11 to 13 range, and only 1 article scored 9. We deemed the quality of these cross sectional studies did not affect the quality of our meta-analysis.

### Statistical Analysis

If values for the Pearson correlation coefficient (r) were not available in a particular study, the Spearman correlation coefficient (r_s_) or regression coefficient (b) with the corresponding 95% confidence interval (CI) were used to estimate r values according to the following formulas: r = 2 × sin(r_s_×π/6) [12] or t = b/SE_b_ (the standard error of b), r^2^ = t^2^/(t^2^+n-2), r×b≥0 [13]

Fisher’s z transformation was performed to convert each correlation coefficient in to an approximate normal distribution [14]. The transformation of r values to Fisher’s z is given by: z = 0.5 × [ln(1+r)-ln(1-r)]. The variance of z is: Vz = 1/ (n-3). The standard error of z is: SEz = 1/√ (n-3) [14]

Meta-analysis of z values were completed using Stata software, version SE 12.0 (Stata Corp LP, TX). Data from the various studies were pooled using the random-effects model, after appropriate conversion. The heterogeneity of the z values between studies was determined by using the chi-square based Q test and I^2^ test [15]. Notable heterogeneity is indicated by an I^2^ value >50% [14]. Next, sensitivity, subgroup and regression analysis were performed, seeking potential sources of heterogeneity. Sensitivity analysis was performed by comparing the results from the random-effects model with those from the fixed-effects model. In a subgroup analysis, studies were stratified by the median PBDEs levels in each study. Meta regression was used to assess the contribution rate of the heterogeneous sources. The Begg’s test and Egger’s test were used to assess the extent of publication bias. The quality of our research reporting was improved by the Preferred Reporting Items for Systematic Reviews and Meta-analyses statement (PRISMA) [16].

## Results

Our initial search yielded 554 potential articles. Of these articles, 525 were excluded after reviewing their abstracts, due to non-relevance (n = 389) or duplications (n = 136). Thirteen articles were excluded due to various reasons (S1 File.). Finally, 16 published articles met the inclusion criteria [17–32]. Some articles included more than one study; therefore, in total, 19 studies were analyzed.

### The correlation between PBDEs exposures and TSH serum levels

**Fig 2:**
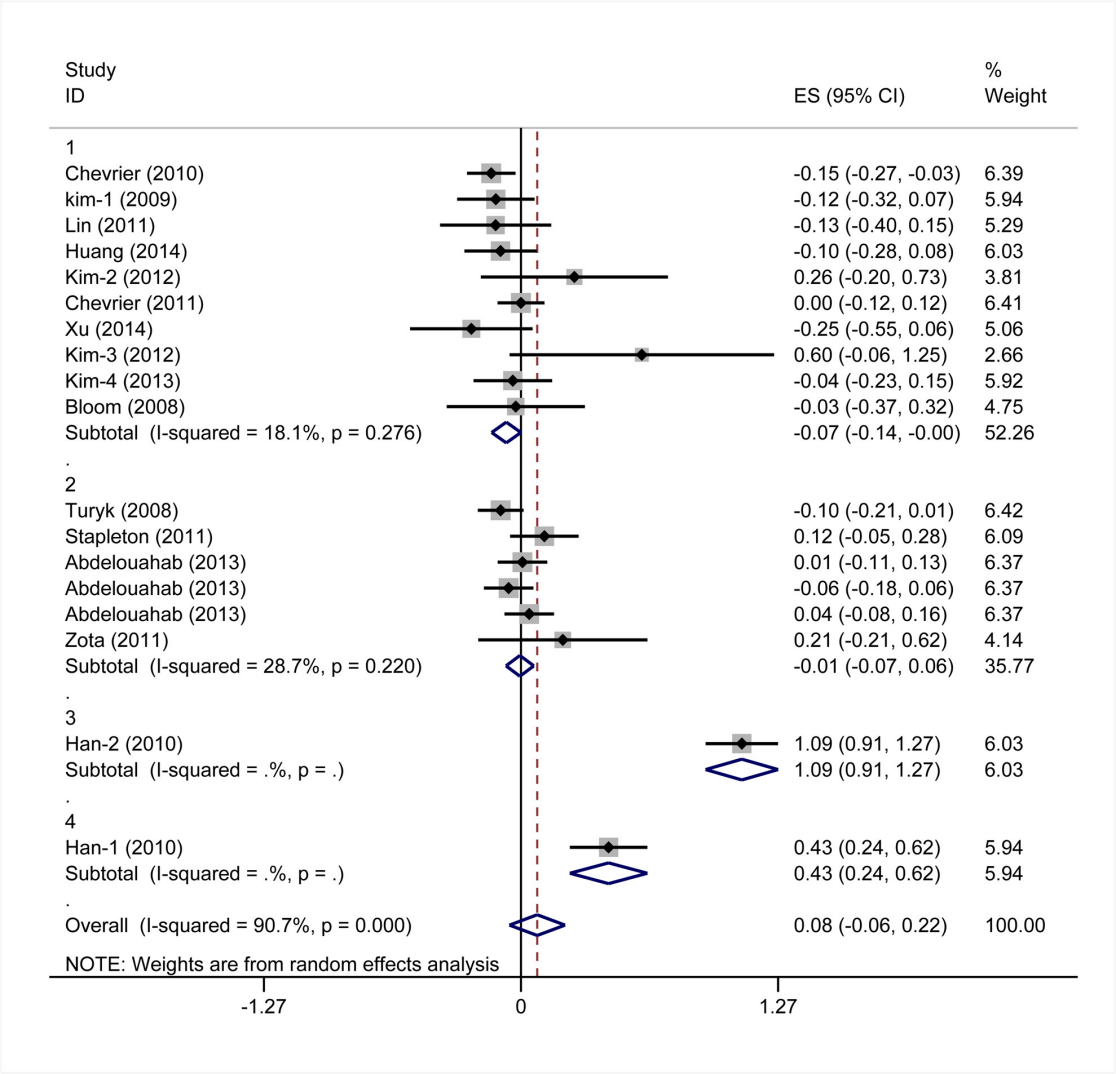
Forest plots of the summary z value with corresponding 95% CIs for the correlation between PBDEs and TSH. (the random-effects model).

**Fig 3:**
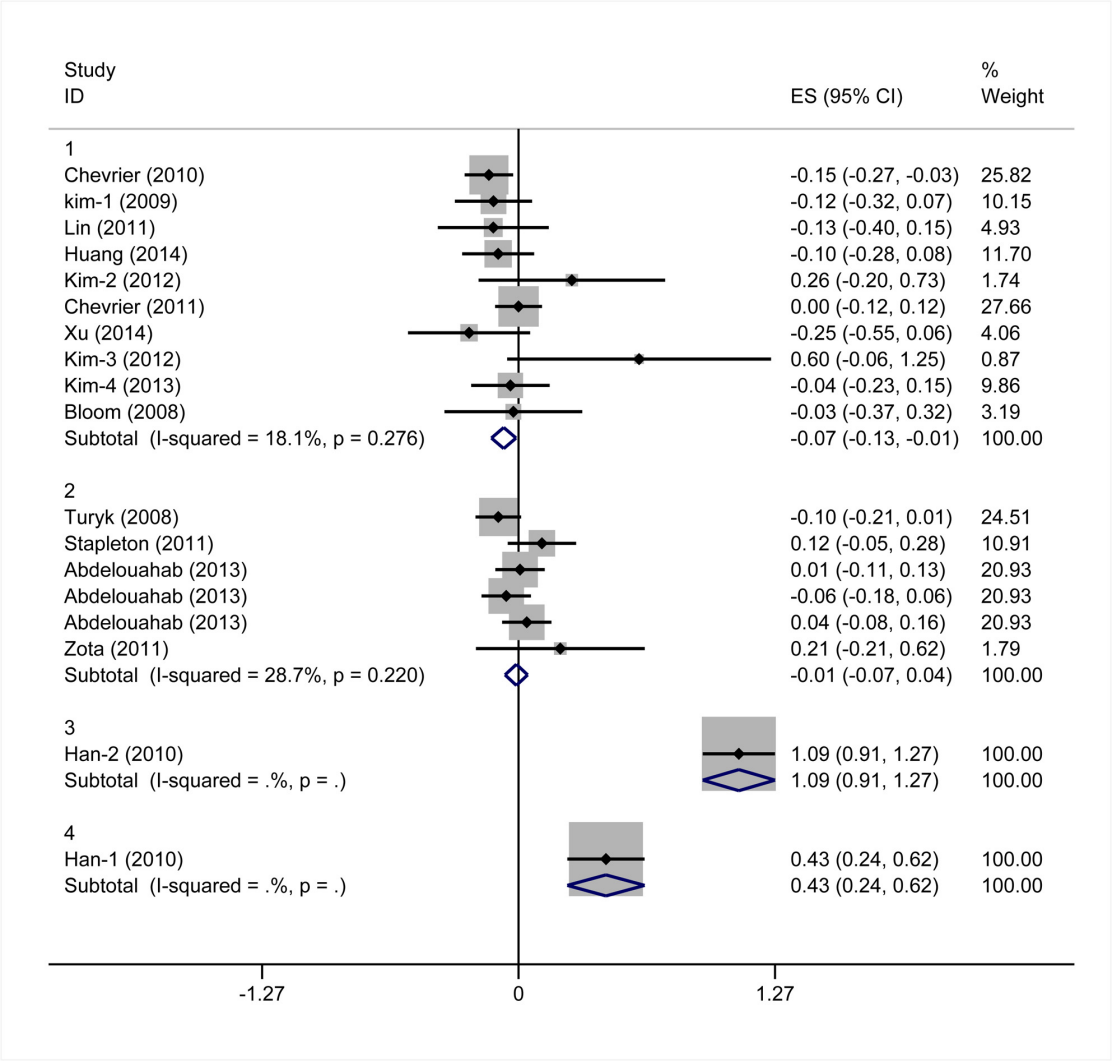
Forest plots of the summary z value with corresponding 95% CIs for the correlation between PBDEs and TSH. (the fixed-effects model).

**Fig 4:**
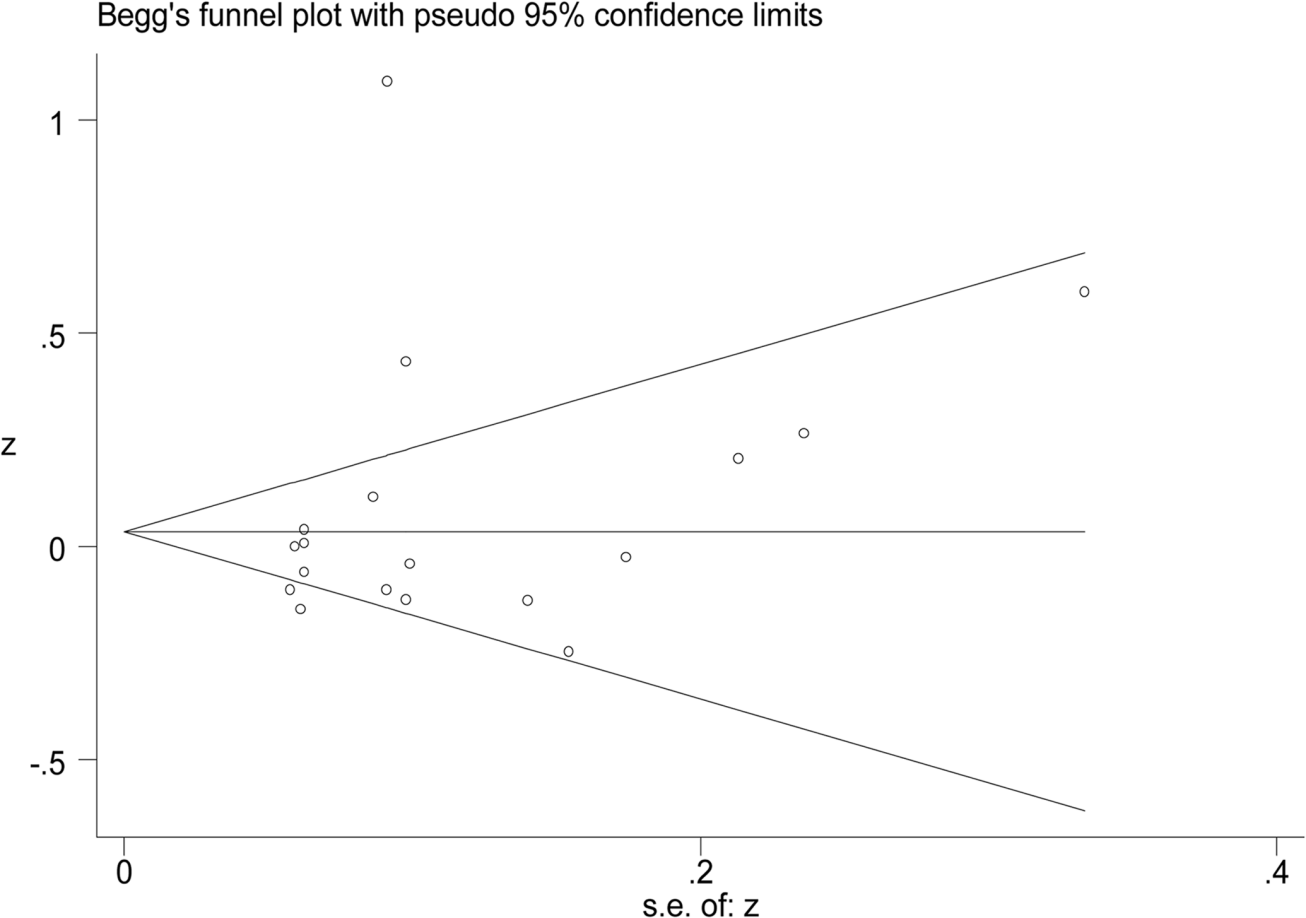
The Begg’s funnel plot of the publication bias. The results indicated no significant public bias (P = 0.068).

Eighteen studies provided data suitable for meta-analysis of correlations between PBDEs exposures and TSH serum levels. The pooled z for all studies (Fig 2) was 0.08 (95% CI -0.06, 0.22), and exhibited notable heterogeneity (I^2^ values = 90.7%). In a subgroup analysis, studies were stratified by the median level of PBDEs in each study. Subgroup one is comprised of studies with median PBDEs levels < 30 ng/g lipids. The pooled z value of subgroup one was -0.07 (95%CI -0.14, 0.00) Subgroup two is comprised of studies with median PBDEs levels between 30 ng/g and 100 ng/g lipids. The pooled z value of subgroup two was -0.01 (95% CI, -0.07, 0.06). Subgroup three is comprised of studies with median PBDEs levels between 100 ng/g and 500 ng/g lipids. The pooled z value of subgroup three was 1.09 (95% CI, 0.91, 1.27). Subgroup four is comprised of studies with median PBDE levels > 500 ng/g lipids. The pooled z value of subgroup four was 0.43 (95% CI, 0.24, 0.62). The I^2^ value of each subgroup was below 30%, and the median difference of PBDEs exposures in different studies, accounted for 46.34% of the between studies variance. The results from each subgroup, using the random-effects model, were consistent with those using the fixed-effects model (Fig 3). The results of Begg’s test and Egger’s test showed no obvious evidence of publication bias (P>0.05). (Fig 4)

### The correlation between PBDEs exposures and TT4 serum levels

**Fig 5:**
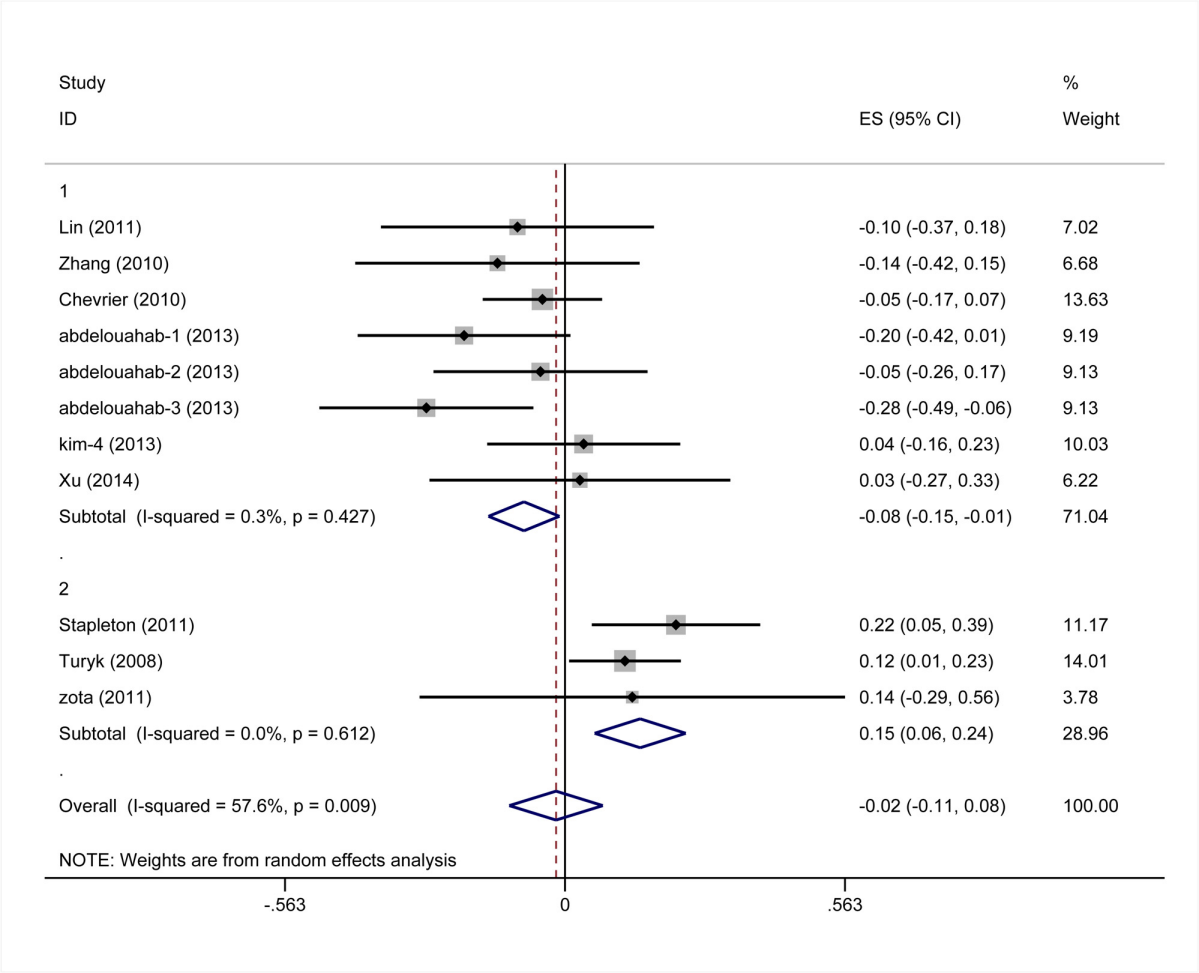
Forest plots of the summary z value with corresponding 95% CIs for the correlation between PBDEs and TT4. (the random-effects model).

**Fig 6:**
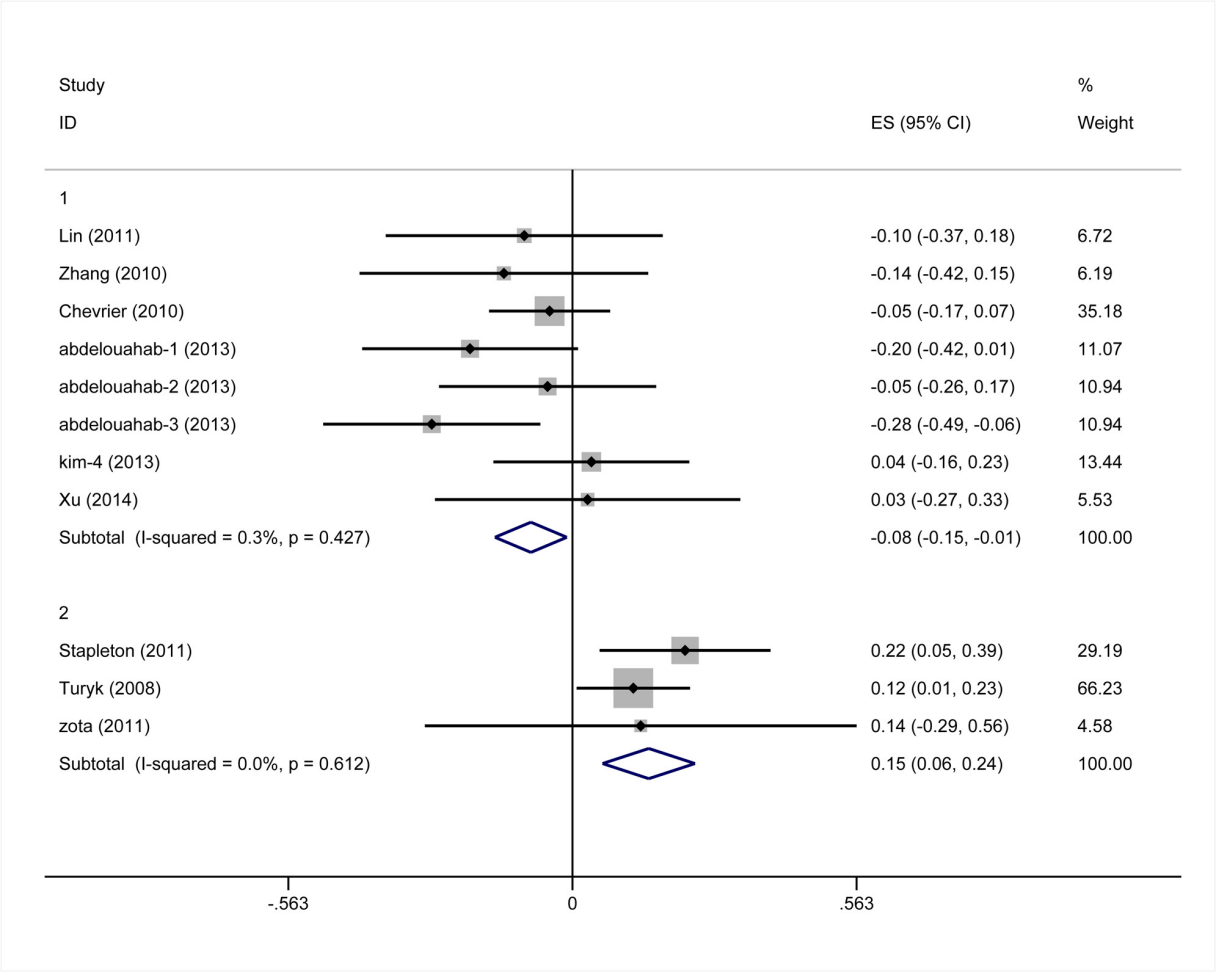
Forest plots of the summary z value with corresponding 95% CIs for the correlation between PBDEs and TT4. (the fixed-effects model).

**Fig 7:**
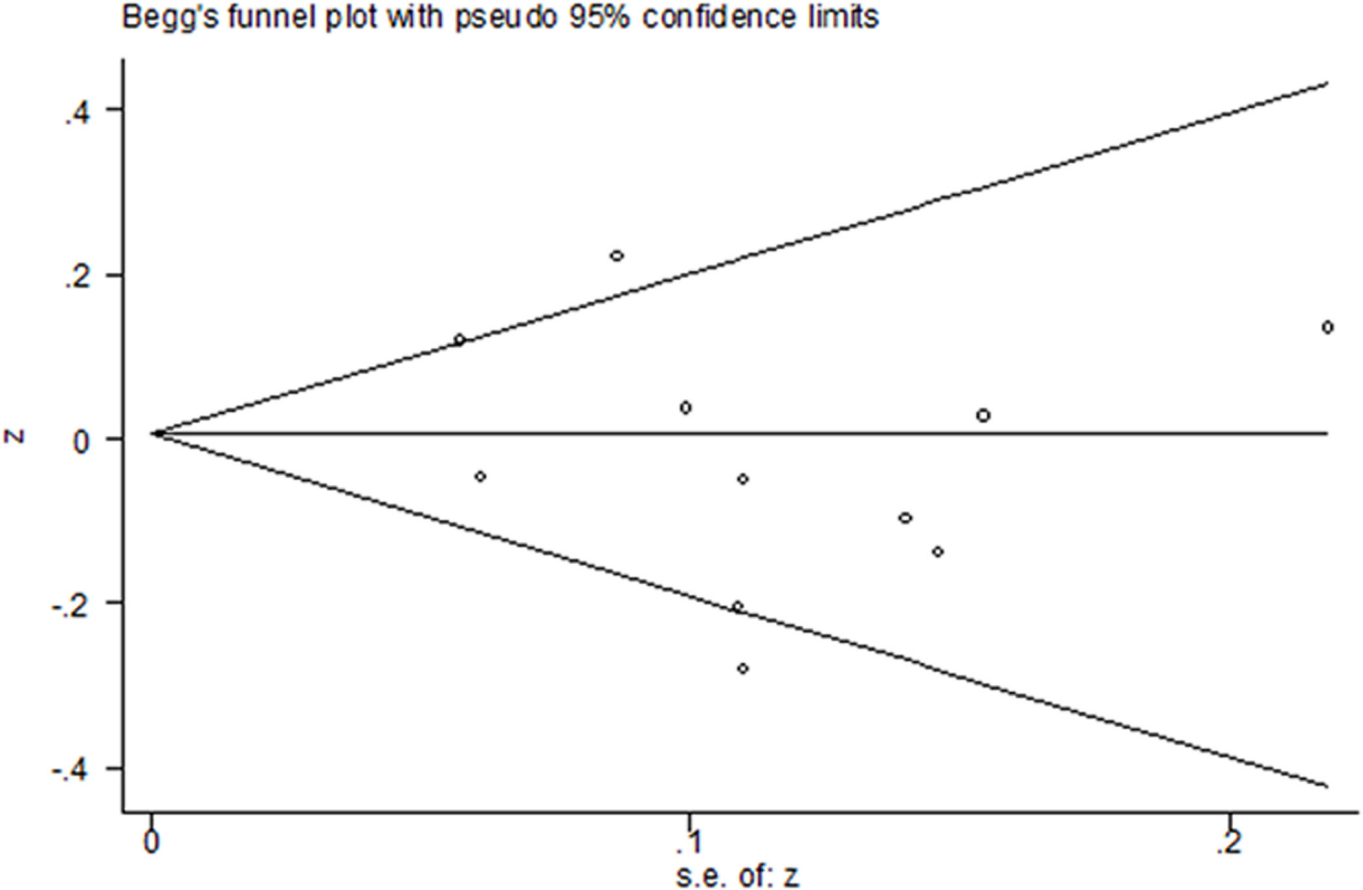
The Begg’s funnel plot of the publication bias. The results indicated no significant public bias (P = 0.696).

Eleven studies provided data suitable for a meta-analysis of correlations between PBDEs exposures and TT4 serum levels. For 6 of these studies, the r values were calculated based on the b values and the 95% CI values of b values provided in the papers. The pooled z value, for all studies, was -0.02 (95% CI, -0.11, 0.08), and exhibited notable heterogeneity (I^2^ values = 57.6%) (Fig 5). In a subgroup analysis, studies were stratified by the median levels of PBDEs exposures in each study. Subgroup one is comprised of studies with median PBDEs levels of < 35 ng/g lipids, and the pooled z value was -0.08(95% CI, -0.15, -0.01). Subgroup two is comprised of studies with median PBDEs levels between 35 ng/g and 100 ng/g lipids, and the pooled z value was 0.15 (95% CI, 0.06, 0.24). The I^2^ values of each subgroup was 0, and the median difference of PBDEs in different studies accounted for 100% of the between studies variance. The results of each subgroup using the random-effects model were consistent with those using the fixed-effects model (Fig 6). The results of Begg’s test and Egger’s test showed no obvious evidence of publication bias (P>0.05) (Fig 7).

## Discussion

PBDEs are ubiquitously found in various kinds of foods and human tissues [33–34]. Respiratory inhalation, dust ingestion, or diary intake may lead to PBDEs exposure, and the compounds can accumulate in a fetus, by crossing the placental barrier [35]. In addition, it has been reported that thyroid gland activity and thyroid hormones metabolism can be disturbed by exposure to PBDEs [36], but the relationship between PBDEs exposures and TH serum levels is still poorly defined, with several studies presenting conflicting findings [21–22, 31,37].The sample sizes of these epidemiological studies were not large enough to detect potentially subtle effects from PBDEs. For enlarging sample size, meta-analysis should be a rational approach.

To our knowledge, our research is the first meta-analysis to determine the association between PBDEs exposure and thyroid function in the general population. In China, several studies [21,38] of individuals exposed to high levels of PBDEs presented remindful results. PBDEs may have different effects on the thyroid system at different levels, and the duration of exposure to PBDEs may also be a factor [25, 31]. Our meta-analysis illustrated complex correlations between PBDE exposures and TSH serum levels, in that a negative correlation presented when the median levels of PBDEs were <30 ng/g lipid, there was no correlation if the median levels of PBDEs were between 30 ng/g and 100 ng/g lipids, and a positive correlation presented if the median levels of PBDEs were > 100 ng/g lipids. A similar correlation pattern between PBDEs exposures and TT4 serum levels was also observed.

We preliminarily estimated a crucial point along this continuum may at PBDEs levels between 30 and 100 ng/g lipids. We determined that the relationship between PBDEs exposures and TH levels probably does not fit a simple mono-phasic curve, but possibly fits a u-shaped curve, thus implying complex mechanisms and chronic processes are in flux. With evidence from the following studies, we speculate that there are two mechanisms resulting in the same endpoint outcome. One mechanism generally leads to an acute and direct effect where data may fit as a mono-phasic curve. The second mechanism leads to a chronic and indirect effect where data may fit an inversed u-shaped curve. Both of them were synthesized to the curve of integral effect.

A previous vitro study [10] had proved that hydroxylated PBDEs congeners displace T4 from transthyretin (TTR). The hydroxylated PBDEs metabolites, which are structurally similar to thyroid hormones, compete with the T4-TTR [39], thus lowering the half-life of T4 in the body [40]. Another approach responsible for thyroid hormone depletion is the induction of phase Ⅱ UDPG enzymes in the liver and subsequent biliary elimination of thyroxine as T4-glucronide [41]. Hallgren and Darnerud demonstrated a small increase in T4-gluronidation following exposure to BDE47 [42]. PBDEs have also been shown to bind to thyroid hormone receptors (TRs) and may have selective effects on thyroid hormone receptor functions [43]. This proposed mechanism may partially explain the direct TT4 lowering effect of PBDEs.

The interaction between rT3 and PBDEs indicates that thyroid hormone deiodinases, which play a key role in the control of T3 cellular levels, might be affected by PBDEs exposures [44]. The D2 deiodinase converts T4 to T3 by removing an iodide from the outer ring of thyroid hormones, whereas D3 deiodinase converts T4 to rT3 by removing an iodide from the inner ring. Finally, D1 can remove iodide from either the inner or outer rings of thyroid hormones. For instance, elevated T4 and rT3 levels appear in mice with D1 mutations, where no changes in T3 or TSH were observed [45]. Elevated T4 and TSH levels appear in mice carrying deletion mutations in D2, with no changes in T3 levels observed [46]. Hypothyroidism, with decreased T4 and T3 levels occur in mice with D3 mutations, with no change in TSH levels observed [47]. Therefore, if PBDEs and/or their metabolites inhibited deiodinase activity, a decrease in T3 levels and an increase in circulating T4 levels should be observed. Increased thyroglobulin antibodies were found in 31% of participants, with the highest PBDEs body burdens, in one study [20], and thyroglobulin antibodies are found in most of patients with Grave’s disease and chronic autoimmune thyroiditis[20]. PBDEs exposure may be the underlying cause of a susceptibility to autoimmune thyroiditis. The findings listed above imply a chronic process in thyroid disease, induced by an indirect mechanism, such as autoimmune responses or mutations.

In an animal study [48], pregnant Wistar rats were administered low dose (140 or 700 ug/kg body weight) of PBDE-47 on gestation day 6 and the offspring of these pregnant Wistar rats were observed for histologic changes of thyroid. Follicular cyst formed in thyroid gland could be found occasionally in the group of 140ug and mild cyst was formed in the group of 700ug. Degenerated follicular epithelium was found in multiple areas in the groups of 140ug and 700 ug, and detachment of thyroid follicular epithelial cells was also found in the colloid. In another animal study [49], Post-mortem examinations were performed to explore potential relationship between exposure to pollutants such as polychlorobiphenyls (PCBs), DDT, toxaphene, PBDEs and thyroid function (TF) in harbor porpoises. Thyroid tissue samples were also collected for immunohistological and histological investigations [49]. The exposure level of principal components, which contained PCBs, PBDEs and DDT, was positively correlated to the proportion of connective tissue and negatively with that of follicle tissue [49]. These results indicate that PBDEs exposures may cause interfollicular fibrosis in the thyroid glands and eventually a loss of thyroid function.

Adverse health effects may differ dependent of serum levels of PBDEs and a variety of other factors, such as race, age, and gender. For example, several studies indicate that newborns and infants, prenatally exposed to high levels of PBDEs, are more vulnerable to multiple adverse effects [50–51]. A recent systematic review [52] has listed and summarized the findings from nine studies about the relationship between PBDEs exposures and thyroid functions without statistical analysis of those data. We have recognized the difference in PBDEs exposure levels and the population involved among those studies. So we specially performed a meta-analysis of correlation coefficient, which is a relative indicator of the relationship between thyroid functions and PBDEs exposures. To minimize the heterogeneity in the meta-analysis, we have further stratified those studies by PBDEs exposure levels and also tried to perform separate analysis in the subgroups of population (i.e.pregnant women, neonates and children). However, there were no enough eligible studies in either of those subgroups like neonates, children and non-pregnant adults after stratified by PBDE exposure levels. A similar correlation pattern was found in pregnant women to that of analysis in all participants (S1 and S2 Figs). There are not enough eligible studies for a separate analysis of each gender as yet. For example, there is only one individual study about PBDEs exposure in men available and no separate study on PBDEs exposure in non-pregnant women.

In this analysis, we included all eligible studies using either peripheral blood or cord blood. The correlation coefficients (r value and r_s_ value) are measures of direction and strength of the linear relationship between two variables, which is not an indicator for the absolute serum or exposure levels. In this meta-analysis, we try to figure out the potential relationship between PBDEs exposures and thyroid function, but not focus on the changes of serum PBDE levels and thyroid hormone levels. Thus, we pooled the all correlation coefficients got from various populations, and performed an combined analysis on the whole. The levels of TH and TSH in cord blood have been widely used to assess the thyroid functions of neonates [53, 54], which could reflect the alterations resulted from intrauterine PBDE exposure. So, it should be considered as a rational way to predict the hazard of PBDEs exposure by pooling the effect sizes of limited studies.

PBDEs congeners with lower bromine contents are particularly persistent, with estimated half-lives ranging from 2 to 12 years, in humans [17]. PBDEs serum levels are a combined effect of exposed intensity and duration. Thus, we considered studies where the same PBDEs serum levels were present generally similar period of the same process. Although the process could not be demonstrated in each cross sectional study, we can observe the process in our meta-analysis based on the levels of median PBDEs exposure in each study.

A meta-analysis of exposure levels to PBDEs [55] indicated an alarming increase of PBDEs levels in the environments and human bodies. The main PBDE congeners were PBDE-47, PBDE-99, PBDE-100 and PBDE-153 for human exposure. In this study, we extracted data from the 16 included articles and performed separate analysis for the correlation between single PBDE congener exposure and thyroid function. We observed a similar correlation pattern for single PBDE congener, such as PBDE-47(S3 Fig) and PBDE-99(S4 Fig), to that of PBDE congener mixture.

In order to complete our analysis, we had to include enough studies in which r values could be extracted or estimated through data conversion of rs or b values. We found that the estimate values were similar to the given values, in a given study [22]. Therefore, we considered the statistical errors of estimated values were too minor to distort real results. The studies where participants experienced exposures to high levels of PBDEs, especially median levels of PBDEs > 100 ng/g lipids, are considerable rare. Therefore, our findings have limitations where exposures to high levels of PBDEs are insufficient for a comprehensive analysis.

Altogether, the findings in this meta-analysis indicate the effects of PBDEs on thyroid function may mainly depend on PBDEs exposure and their serum levels. The relationship between PBDEs exposure and changes in thyroid function seems to fit a approximate u-shaped curve, which await further verification in a proposed longitudinal study. In addition, other factors, such as race, gender, and age may also influence one’s susceptibility to PBDEs. All these factors may have led to conflicting results in various epidemiological studies, which should be taken into account in future studies.

## Supporting Information

S1 PRISMA Checklist PRISMA 2009 checklist. (PDF)

S1 Fig Forest plots for the correlation between PBDEs and TSH. (TIF)

S2 Fig Forest plots for the correlation between PBDEs and TT4. (TIF)

S3 Fig Forest plots for the correlation between BDE-47 and TT4. (TIF)

S4 Fig Forest plots for the correlation between BDE-99 and TSH. (TIF)

S1 File The 13 full-text excluded articles and reasons for the exclusion. (PDF)
