# Supplementary figures and images for: The Correlation between Polybrominated Diphenyl Ethers (PBDEs) and Thyroid Hormones in the General Population: A Meta-Analysis
Source: PLoS One. 2015 May 18;10(5):e0126989. doi: 10.1371/journal.pone.0126989 (PMC4436299; doi:10.1371/journal.pone.0126989)

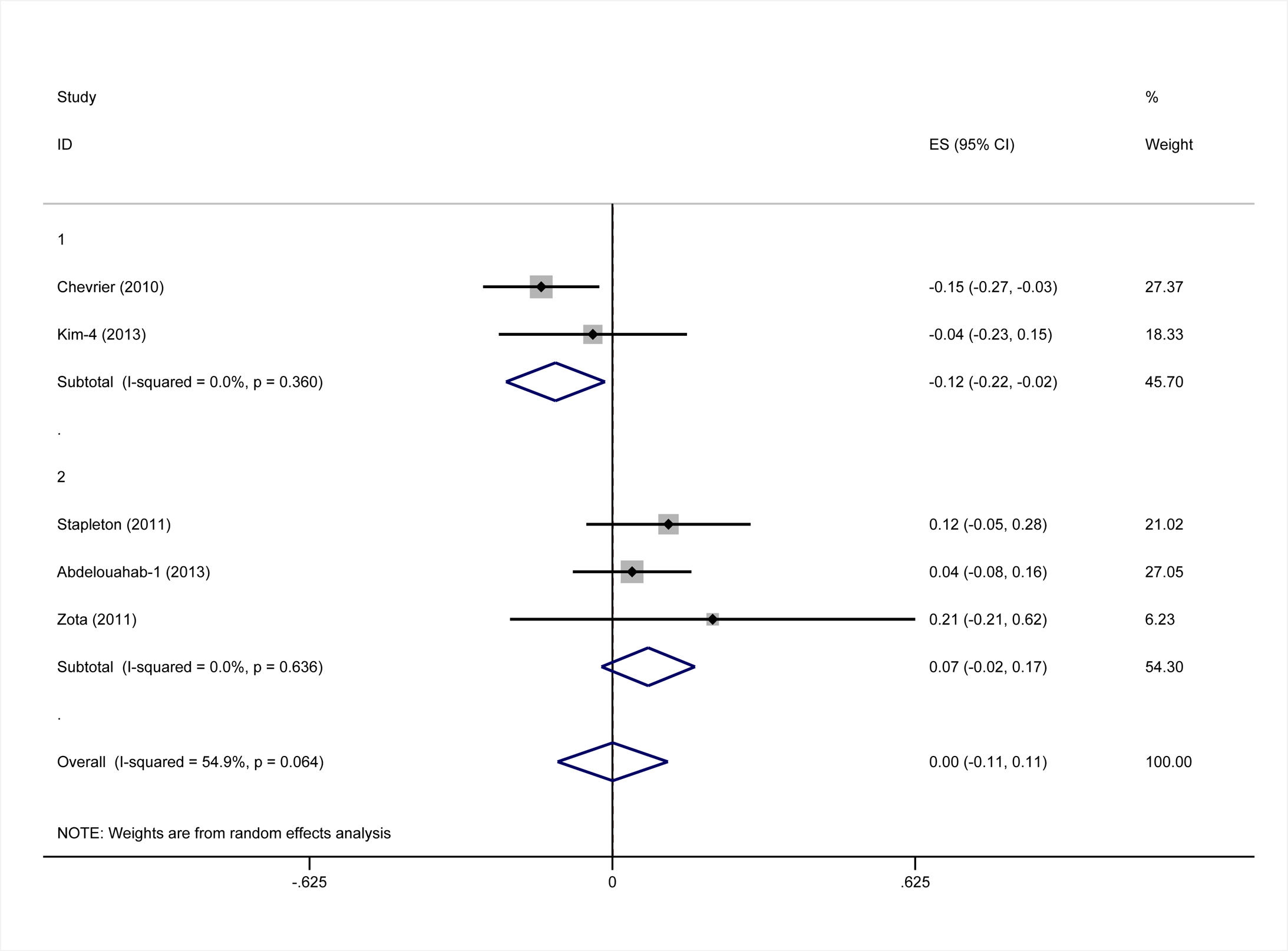

Supplement: S1 Fig — (TIF) [file pone.0126989.s002.tif]

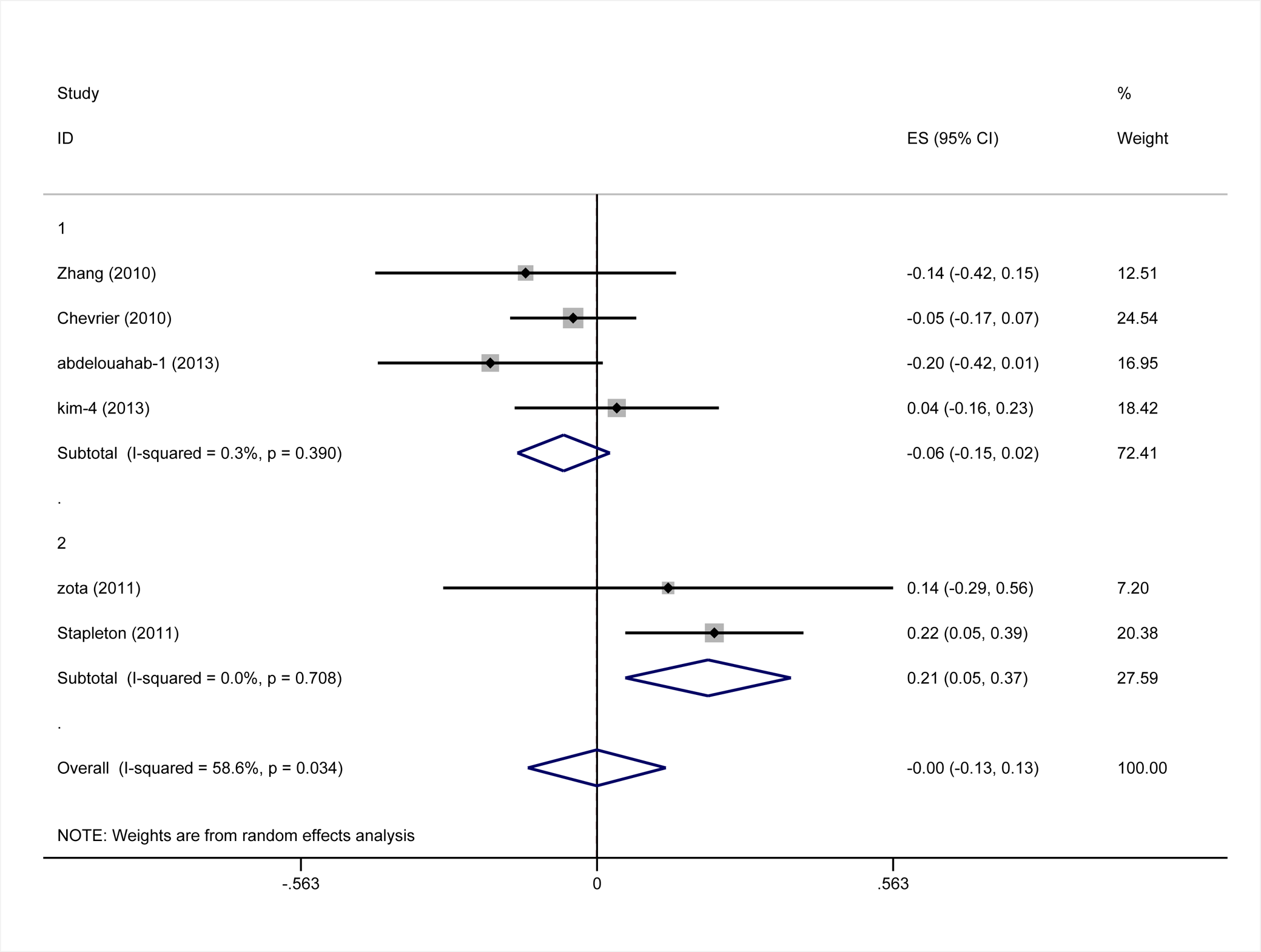

Supplement: S2 Fig — (TIF) [file pone.0126989.s003.tif]

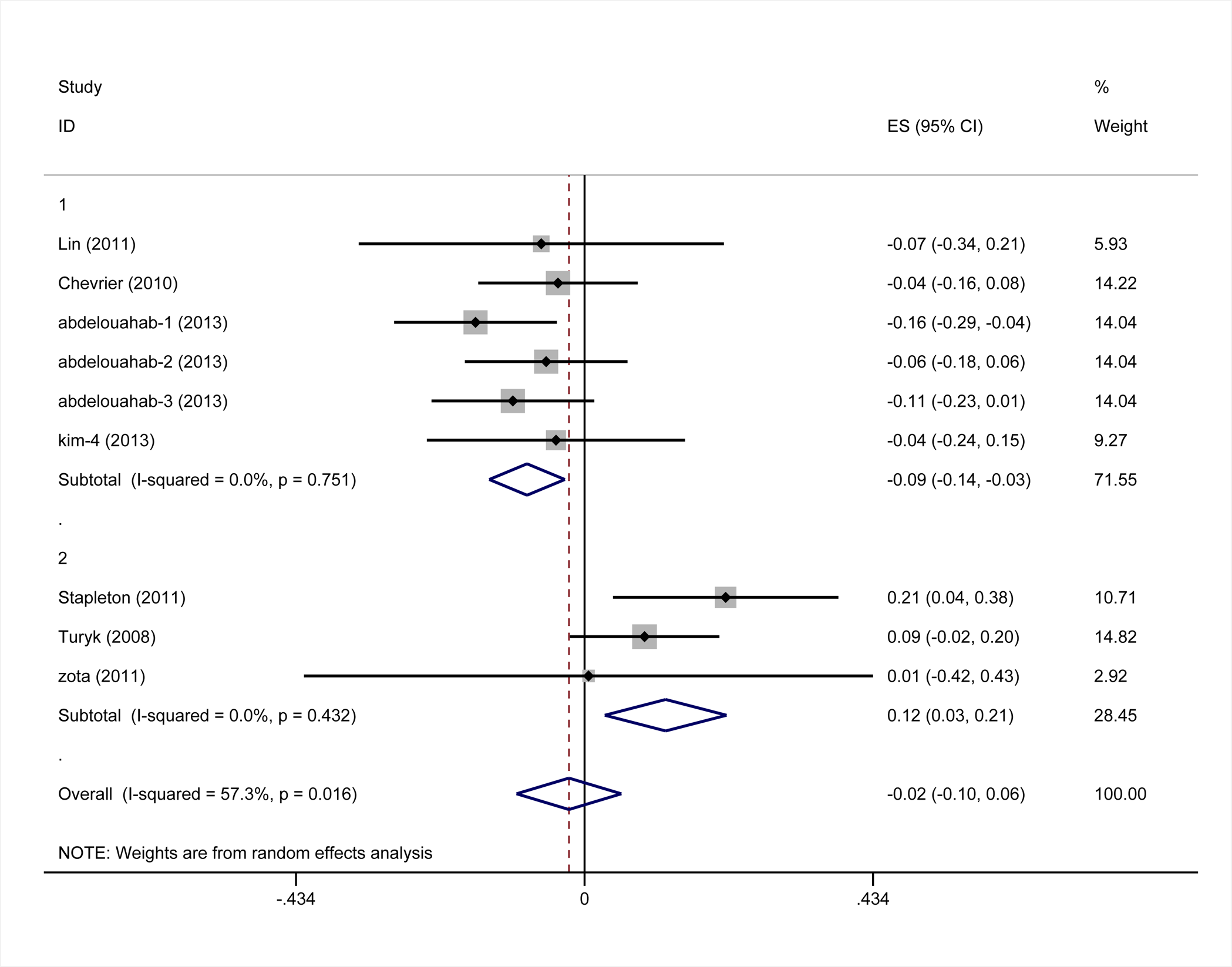

Supplement: S3 Fig — (TIF) [file pone.0126989.s004.tif]

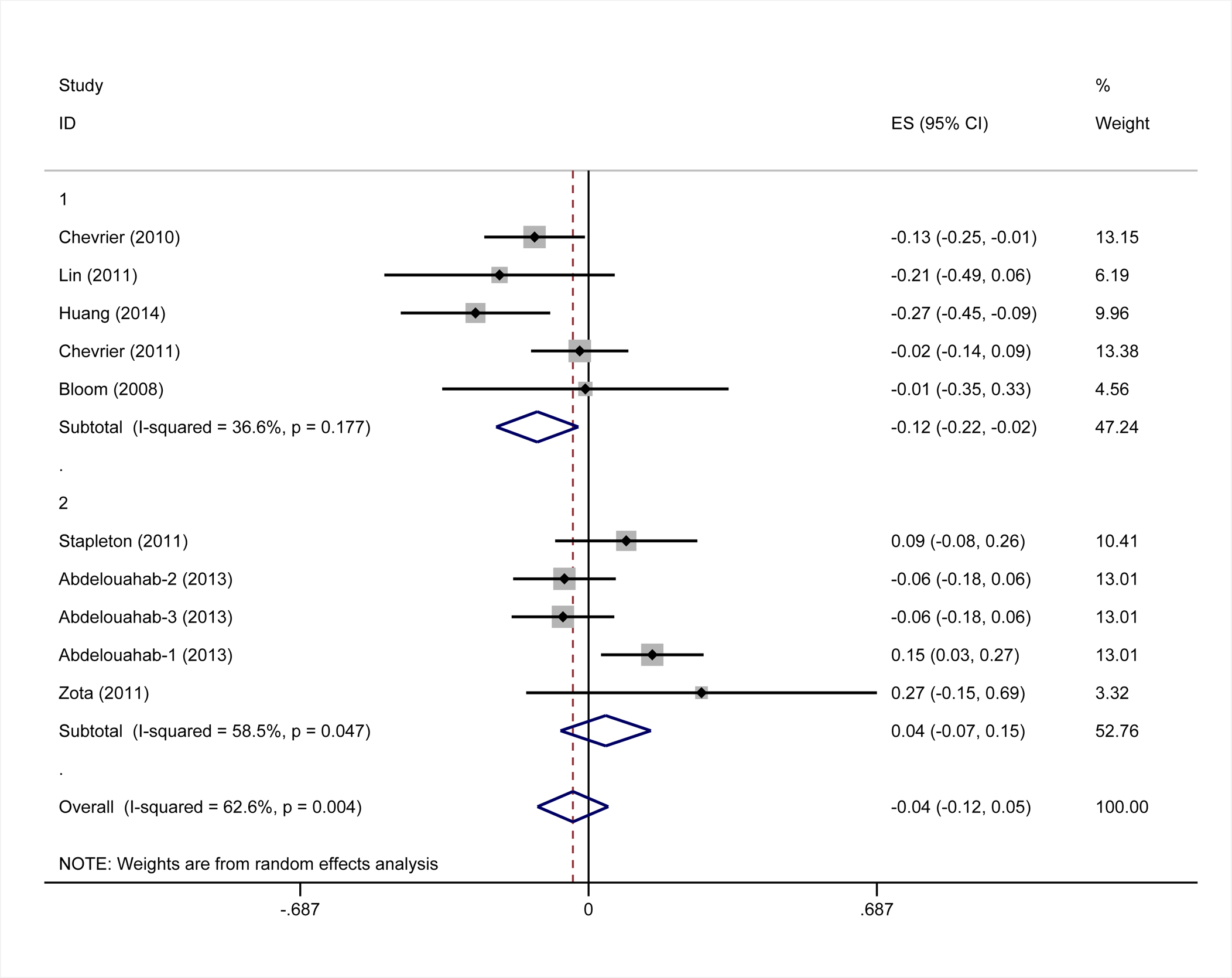

Supplement: S4 Fig — (TIF) [file pone.0126989.s005.tif]
