# Supplementary material for: The Correlation between Polybrominated Diphenyl Ethers (PBDEs) and Thyroid Hormones in the General Population: A Meta-Analysis
Source: PLoS One. 2015 May 18;10(5):e0126989. doi: 10.1371/journal.pone.0126989 (PMC4436299; doi:10.1371/journal.pone.0126989)
Supplement: S1 File — (PDF) [file pone.0126989.s006.pdf]

The 13 full-text excluded articles and reasons for the exclusion

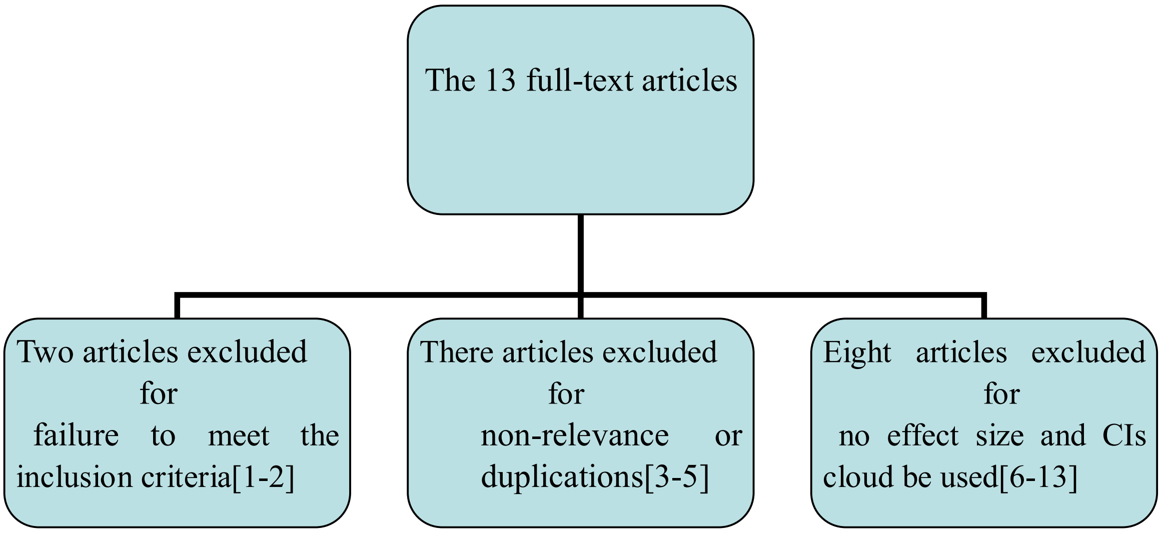

References

1. Shy CG, Huang HL, Chao HR, Chang-Chien GP. Cord blood levels of thyroid hormones and IGF-1 weakly correlate with breast milk levels of PBDEs in Taiwan. International journal of hygiene and environmental health. 2012;215(3):345-51.
2. Eggesbo M, Thomsen C, Jorgensen JV, Becher G, Oyvind Odland J, Longnecker MP, et al. Associations between brominated flame retardants in human milk and thyroid-stimulating hormone (TSH) in neonates. Environmental Research. 2011;111(6):737-43.
3. Goodman JE, Kerper LE, Johnson GT, Harbison RD, Cordero R, Lee RV, et al. PBDE flame retardants and thyroid hormones during pregnancy. Environmental health perspectives. 2010;118(12):a520; author reply a-1.
4. Darnerud PO, Eriksen GS, Johannesson T, Larsen PB, Viluksela M. Polybrominated diphenyl ethers: occurrence, dietary exposure, and toxicology. Environmental health perspectives. 2001;109 Suppl 1:49-68.
5. Han G, Ding G, Lou X, Wang X, Shen H, Zhou Y. Study of between persistent organic pollutants (POPs) content in children vein blood and thyroid stimulating hormone (TSH) in the capcitors dismantlement area. Wei sheng yan jiu = Journal of hygiene research. 2010;39(5):580-2.
6. Buttke DE, Wolkin A, Stapleton HM, Miranda ML. Associations between serum levels of polybrominated diphenyl ether (PBDE) flame retardants and environmental and behavioral factors in pregnant women. Journal of exposure science & environmental epidemiology. 2013;23(2):176-82.
7. Dallaire R, Dewailly E, Pereg D, Dery S, Ayotte P, Author A, et al. Thyroid function and plasma concentrations of polyhalogenated compounds in inuit adults. Environmental Health Perspectives. 2009;117(9):1380-6.
8. Gascon M, Vrijheid M, Martinez D, Forns J, Grimalt JO, Torrent M, et al. Effects of pre and postnatal exposure to low levels of polybromodiphenyl ethers on neurodevelopment and thyroid hormone levels at 4 years of age. Environment

international. 2011;37(3):605-11.
9. Kim UJ, Lee IS, Kim HS, Oh JE. Monitoring of PBDEs concentration in umbilical cord blood and breast milk from Korean population and estimating the effects of various parameters on accumulation in humans. Chemosphere. 2011;85(3):487-93.
10. Li MY, Jin J, Yang CQ, Wang Y, Ding WW, Yang XF, et al. [Correlations between PBDEs and thyroid hormone concentrations in adults from production source area]. Huan Jing Ke Xue. 2011;32(11):3271-6.
11. Mazdai A, Dodder NG, Abernathy MP, Hites RA, Bigsby RM. Polybrominated diphenyl ethers in maternal and fetal blood samples. Environmental health perspectives. 2003;111(9):1249-52.
12. Roze E, Meijer L, Bakker A, Van Braeckel KN, Sauer PJ, Bos AF. Prenatal exposure to organohalogens, including brominated flame retardants, influences motor, cognitive, and behavioral performance at school age. Environmental health perspectives. 2009;117(12):1953-8.
13. Yard EE, Terrell ML, Hunt DR, Cameron LL, Small CM, McGeehin MA, et al. Incidence of thyroid disease following exposure to polybrominated biphenyls and polychlorinated biphenyls, Michigan, 1974-2006. Chemosphere. 2011;84(7):863-8.
